# Prevalence of health anxiety in indian ophthalmologists during COVID-19: a survey

**DOI:** 10.1192/j.eurpsy.2021.502

**Published:** 2021-08-13

**Authors:** C. K

**Affiliations:** Psychiatry, National Institute of Mental Health and Neuro Sciences, BENGALURU, India

**Keywords:** prevalence, Covid, health anxiety, Ophthalmologists

## Abstract

**Introduction:**

Mental health concerns are common in health care workers during pandemic. There are no studies of the prevalence of health anxiety in ophthalmologists in India.

**Objectives:**

To estimate the prevalence of health anxiety in ophthalmologists practicing in India during the ongoing pandemic.

**Methods:**

A questionnaire-based online survey on the “changes and challenges during COVID-19” using Google forms was sent to all members of the All India Ophthalmological Society. Besides demographics, the survey had questions to assess the general mental and medical health status of the ophthalmologists. Short Health Anxiety Inventory (SHAI) was used to assess health anxiety.

**Results:**

1027 ophthalmologists responded to the study. Higher stress was experience by 83.1% compared to pre-COVID while examining patients closely (35.9%) or during surgery due to the risk of aerosol generation (29.3%). SHAI score >20 was observed in 5.6%. Only emergency services were being provided by 50% and 17% in the SHAI > 20 group were not working as compared to overall 14%.

**Conclusions:**

Our findings indicate that a majority of the ophthalmologists were under stress during the COVID-19 pandemic but only a small proportion experienced health anxiety. It is likely that mental health issues may arise among ophthalmologists in the foreseeable future.

